# Vitiligo, alkaptonuria, and nitisinone—A report of three families and review of the literature

**DOI:** 10.1002/jmd2.12225

**Published:** 2021-06-14

**Authors:** Lakshminarayan Ranganath, Milad Khedr, Leanne A. Evans, Helen Bygott, Emily Luangrath, Elizabeth West

**Affiliations:** ^1^ Department of Clinical Biochemistry and Metabolic Medicine Royal Liverpool University Hospital Liverpool UK; ^2^ Department of Dermatology Royal Liverpool University Hospital Liverpool UK

**Keywords:** alkaptonuria, homogentisic acid, nitisinone, proton‐pump inhibitors, tyrosine, vitiligo

## Abstract

Four patients, from three families, with alkaptonuria receiving 4‐hydroxyphenylpyruvate dioxygenase‐inhibiting nitisinone therapy, which lowers homogentisic acid and increases tyrosine, developed vitiligo. Three of the four patients were receiving nitisinone 2 mg daily, while the fourth was on 10 mg daily. All four patients were either receiving or had received transiently proton‐pump inhibitors as therapy for dyspepsia. The ages of the patients were 35, 42, 40, and 67 years, respectively. Three patients were men and one was a woman. All four patients were either taking a proton‐pump inhibitor or had been taking one at some point. Three of the four were of South Asian and one of Caucasian background. The three patients with South Asian background also had either a personal or family history of autoimmune disease. Distressing vitiligo, initially in an acrofacial distribution, developed unexpectedly in these four patients, before then progressing to involve other parts of the body. Potential factors in the appearance of vitiligo in this setting, including nitisinone and other drug therapy, are explored and responses to the appearance of vitiligo are discussed.

## 1 INTRODUCTION

Alkaptonuria (AKU) (OMIM#203500) is an inherited disorder present from birth, with a frequency of around 1 in 250 000 people in most parts of the world, where consanguinity is not common.^1^, ^2^, ^3^ AKU is characterized by increased accumulation of homogentisic acid (HGA) due to deficient homogentisate 1,2 dioxygenase activity (EC:1.13.11.5).^4^ Accumulating HGA undergoes oxidation to a melanin‐like pigment via a benzoquinone acetate intermediary in a process known as ochronosis, in which the brown‐black pigment deposits in joint and spine cartilage, tendons, and ligaments.^5^, ^6^ The resulting ochronotic tissue is stiff and brittle and is the precursor for the damaging clinical features of the disease.^7^

Multiple organs and tissues are affected in AKU, a severe, progressive, multisystem disease with a delayed onset of symptomatic disease. The involvement of eyes, ears, laryngo‐tracheal‐bronchial tree, articular and fibrocartilage of joints and spine, urinary system, as well as the heart and vasculature has been well described.^8^ The clinical features in AKU, namely kidney and prostate stones, aortic stenosis, bone fractures, tendon/ligament/muscle ruptures, kyphosis, scoliosis, spinal surgery, and joint replacements, reflect the damage to the predisposed tissues.^9^

Until recently, there has been a lack of HGA‐lowering disease‐modifying therapy.^10^ Nitisinone inhibits p‐hydroxyphenyl‐pyruvate dioxygenase enzyme and has been used as treatment for hereditary tyrosinemia type 1 since 1991.^11^ In 1998, it was hypothesized that nitisinone could decrease HGA, and was trialed in the National Institutes of Health, USA, with an inconclusive outcome.^12^ Since 2012, 60 patients have received nitisinone 2 mg daily and followed up for at least 12 months in the United Kingdom National Alkaptonuria Centre (NAC); overall, 88 patients have visited the center at least once. Data on 28 patients, not on nitisinone, have been assessed as part of this article. Nitisinone has been used off‐license under approval from NHS England Highly Specialized Services NAC, subject to annual review of safety and efficacy. As part of the safety and efficacy review, we report four cases of vitiligo, from three families, in patients attending the Royal Liverpool University Hospital which hosts the NAC, after commencing nitisinone therapy. No cases of vitiligo were seen in those not on nitisinone. These cases are described and possible genesis of these lesions are discussed. Ochronosis of the skin and nails in untreated AKU and itchy rash following nitisinone treatment in AKU has been previously described.^13^, ^14^, ^15^

The data on cases 1 to 3, presented below, collected from the NAC was approved by the Institutional Audit Committee (Audit No: ACO3836). Case 4 with AKU participated in the Suitability of Nitisinone in Alkaptonuria 2 (SONIA 2) study (EC Liverpool [NRES Committee North‐West ‐ Liverpool Central] Reference number: 13/NW/0567). Medline search was carried out using search words such as vitiligo, alkaptonuria, nitisinone, and hereditary tyrosinemia type 1.

## 2 CASE REPORTS

### 2.1 Case 1

**FIGURE 1:**
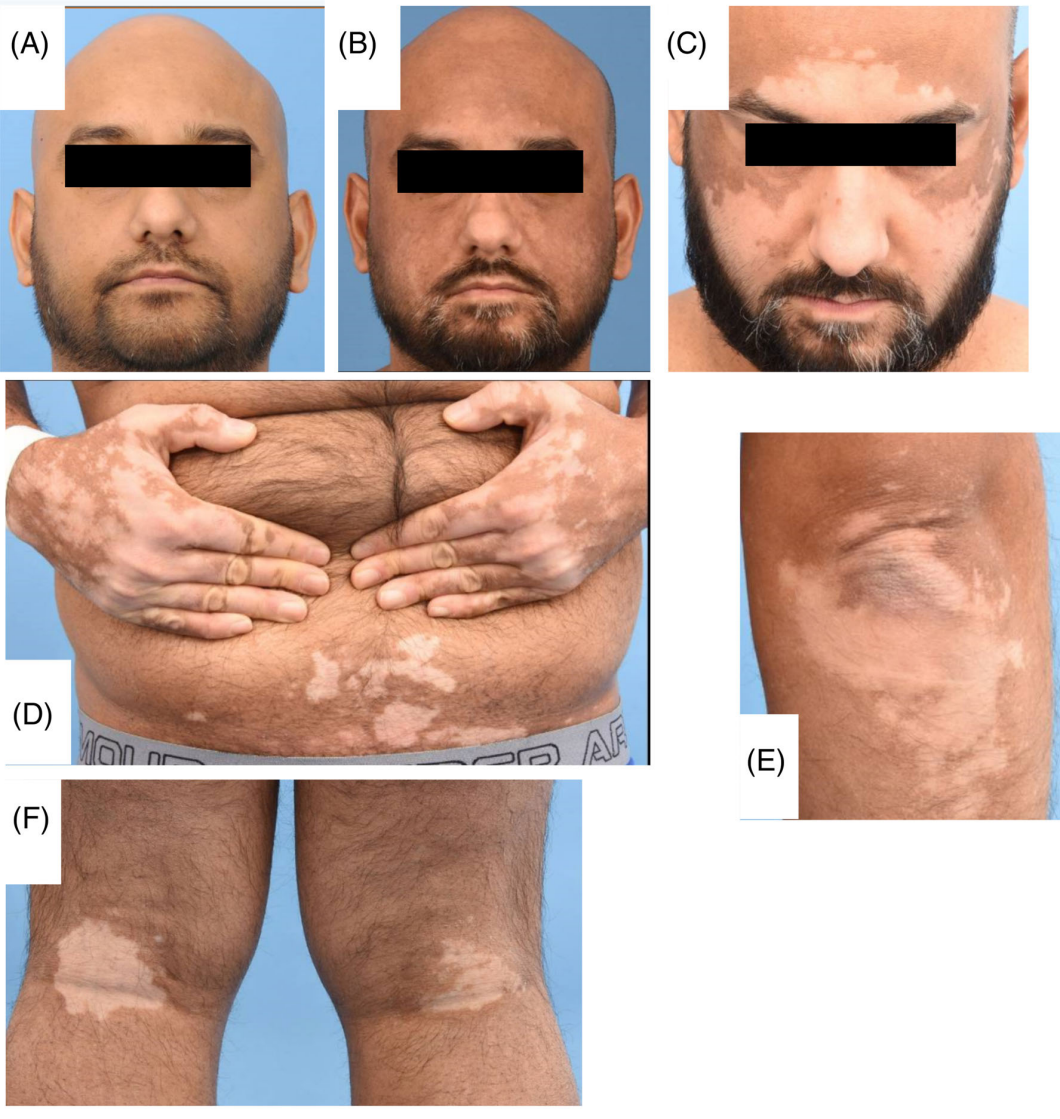
Photographs in case 1 showing (A) normal face pigmentation pre‐nitisinone in 2014, (B) earlier facial vitiligo in 2017 and (C) more extensive vitiligo in 2020, (D) shows hand and trunk vitiligo in 2020, (E) shows vitiligo on extensor surface of elbow in 2020, and (F) shows bilateral popliteal fossa vitiligo in 2020

A 35‐year‐old man of South Asian background presented with recently diagnosed established alkaptonuria characterized by lumbar spondylosis, arthropathy in both feet, left shoulder, mild stable aortic valve stenosis, and mitral valve calcification and regurgitation at the baseline visit (Tables 1 and 2, Figure 1). Out of five brothers and a sister, three brothers (including this patient) and a sister were diagnosed with Alkaptonuria. He mentioned no pigmentary skin changes on questioning at baseline. Physical examination revealed a man of 1.82 m and a weight of 87 kg with a body mass index of 26.5 kg/m^2^. Blood pressure was 125/80. There was no pigmentation of the skin or the nails of the hands or feet. At baseline, he had been taking paracetamol 500 mg and ibuprofen 300 mg when needed. He was commenced on nitisinone 2 mg alternate days for 3 months and then the dose was increased to 2 mg daily. At review 13 months after baseline, he was found to be vitamin D deficient, and was taking lansoprazole 15 mg, lidocaine 5% patches, liquid oral morphine 30 mg, and nitisinone 2 mg. Two years after the baseline visit, he was found to have compensated primary hypothyroidism but had discontinued lansoprazole. Four years after the baseline visit, he was diagnosed with vitiligo and itching affecting his face, hands, and lower abdomen and was using topical steroid creams. He was also taking thyroxine 100 mg daily by this time. His vitiligo had progressed much more by 6 years since the first visit, involving dorsal and palm aspects of his hands, dorsal aspects of his feet, trunk, knees, and popliteal fossa in addition to his face and lower abdomen.

**TABLE 1:** Characteristics of vitiligo in four patients with nitisinone‐treated alkaptonuria

		Case 1	Case 2	Case 3	Case 4
Ethnic background		South Asian	South Asian	South Asian	Caucasian
Age at baseline (y)		35	42	40	67
Sex		Male	Male	Female	Male
Dose of nitisinone		2 mg	2 mg	2 mg	10 mg
Areas affected by vitiligo	Face	Yes	Yes	Yes	No
	Hands	Hands	Yes	Yes	Yes
	Feet	Yes	No	No	Yes
	Trunk	Yes	Yes	Yes	No
	Other	Knees	Genital, scalp	—	—
Onset of vitiligo after nitisinone (mo.)		51	48	36	3
Hyperpigmentation preceding vitiligo		No	Yes (at 12 mo.)	Yes	No
Whether vitiligo accompanied by itching		Yes	Yes	Yes	No
How was itching treated		Topical steroids	Topical steroids	Antihistamine	None
Known autoimmune disorder		Hypothyroidism	No	Hypothyroidism (age 18 y)	No
Family history autoimmune disorder		Sister with SLE	Yes, as with case 1	No	None
Type of vitiligo		Nonsegmental	Nonsegmental	Nonsegmental	Nonsegmental
Progression of vitiligo		Yes	Yes	Yes	Yes
Family history of vitiligo		Yes	Yes	No	No
History of proton‐pump inhibitors therapy		Transient use of Lansoprazole	Omeprazole	Omeprazole	Omeprazole
Timing of proton‐pump inhibitors		Discontinued after brief use	Vitiligo more than 1 y of PPI	Prior to baseline visit	Vitiligo 6 wk post‐PPI

Abbreviation: PPI, proton‐pump inhibitor therapy; SLE, systemic lupus erythematosus.

**TABLE 2:** Baseline^c^ laboratory data in four cases of vitiligo in nitisinone‐treated alkaptonuria

Measurement	Normal ranges	Case 1	Case 2	Case 3	Case 4
Sex		Male	Male	Female	Male
Serum urea (mmol/L)	2.5‐7.8	3.1	4.6	4.2	5.8
Serum creatinine (μmol/L)	50‐130	79	72	62	82
Adjusted serum calcium (mmol/L)	2.2‐2.6	2.37	2.32	2.29	2.5
Total bilirubin (μmol/L)	<21	14	14	7	11
Gamma glutamyl transferase (U/L)	<35 F; <50 M	15	19	36	79
Alkaline phosphatase (U/L)	30‐130	62	73	66	79
Alanine transaminase (U/L)	<35 F; <50 M	21	21	28	22
Plasma glucose (mmol/L)	3.5‐6.0	5.1	5.4	4.6	4.7
FreeT4 (pmol/L)	10‐22	15.6	19.6	22.4	—
TSH (mU/L)	0.3‐6.0	10.3	2.5	0.7	—
Hemoglobin (g/L)	118‐148 F; 130‐160 M	137	134	125	165
White cell count (× 10^9^/L)	3.5‐11	5.7	12.0	4.4	9.3
Platelets (× 10^9^/L)	150‐400	292	356	272	221
Serum homogentisic acid (μmol/L)^a^	<3.1	19.0	22.9	34.1	52.4
Serum homogentisic acid (μmol/L)^b^	<3.1	6.3	7.3	4.5	0.4
Serum tyrosine (μmol/L)^a^	26‐96	56	44	33	80
Serum tyrosine (μmol/L)^b^	26‐96	503	692	997	1394
Increase in tyrosine post‐nitisinone (× baseline)		9.0	15.7	30.2	17.4
Serum nitisinone (μmol/L)^a^	<0.2	<0.2	<0.2	<0.2	<0.2
Serum nitisinone (μmol/L)^b^	<0.2	0.6	0.9	0.8	4.1
24‐h urine homogentisic acid (μmol/d)^a^	NA	17 870	24 611	23 682	42 847
24‐h urine homogentisic acid (μmol/d)^b^	NA	1737	2350	886	92

Abbreviation: NA, not available; TSH, thyroid stimulating hormone.

^a^ Pre‐nitisinone.

^b^ Post‐nitisinone.

^c^ Baseline unless otherwise stated.

### 2.2 Case 2

**FIGURE 2:**
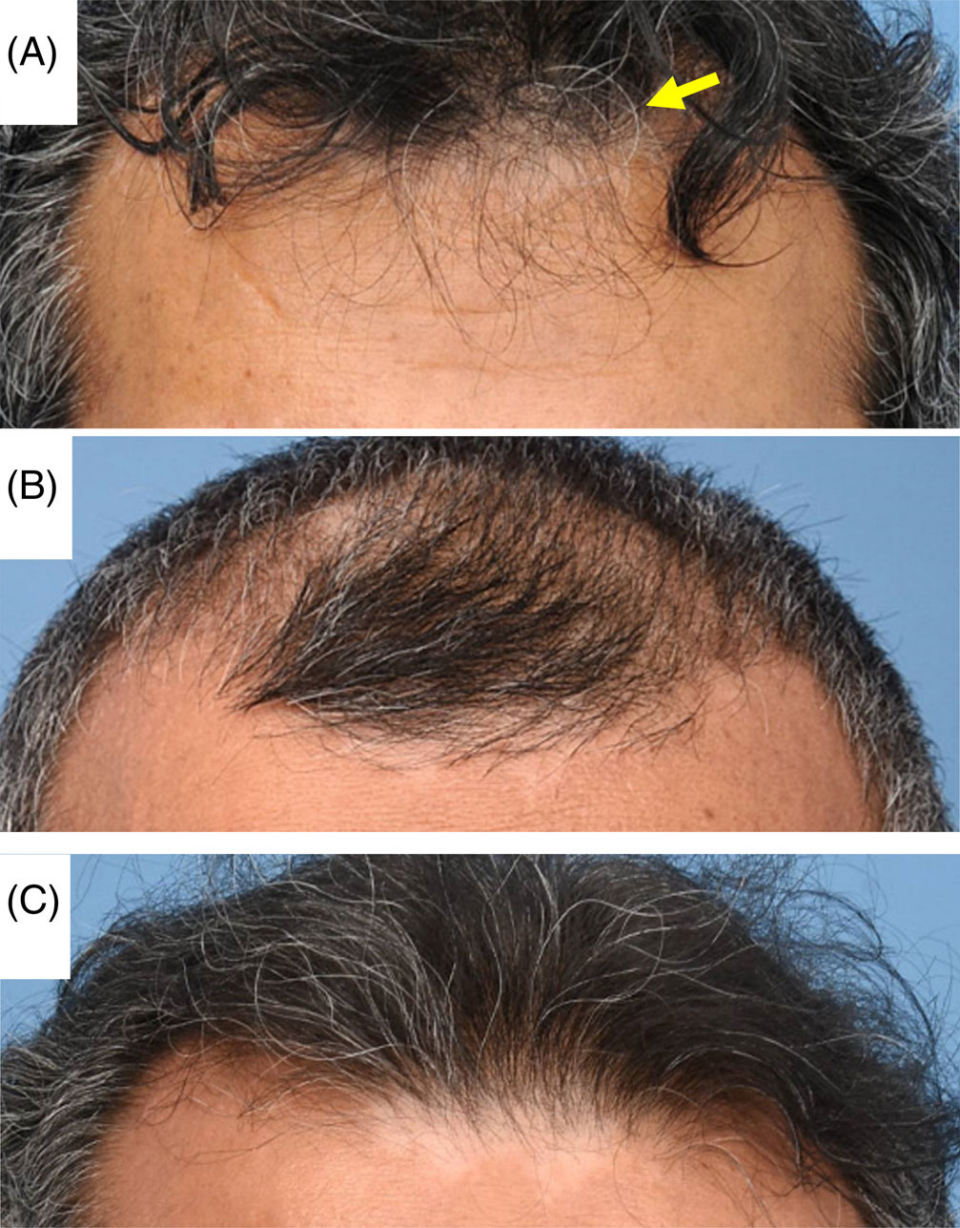
Photographs in case 2 showing vitiligo in forehead and scalp in (A) 2017, (B) 2018, and (C) 2019

A 42‐year‐old man of South Asian background recently diagnosed with AKU was referred for further management at first visit (Tables 1 and 2, Figure 2). He has bilateral ear and eye ochronosis, nail ochronosis, previously passed renal stones at age 27 years, dyspepsia, angina, generalized spondylosis affecting, cervical, thoracic and lumber spine, with generalized joint pains. He is one of the siblings mentioned in case 1. He was 1.67 m and weighing 70 kg, with a blood pressure of 116/74 mm Hg. His medications consisted of buprenorphine patches, co‐codamol, vitamin C, vitamin D, cetirizine, and beclomethasone cream. He was commenced on nitisinone 2 mg alternate days for 3 months and then the dose was increased to 2 mg daily. At the 2‐year visit, an increase in skin pigmentation was noticed in the dorsal aspects of the thumb and middle finger of the right hand. At the 3‐year visit, he mentioned increased discoloration of the skin in the genital region. At his 4‐year visit, he was diagnosed with vitiligo affecting his scalp, periorbital regions, and groin. His sister with AKU also had systemic lupus erythematosus and has not developed vitiligo so far despite also being on nitisinone 2 mg for 5 years. Five years after the baseline visit, the vitiligo had extended to involve his trunk. He was on the following mediations at this time namely, nitisinone 2 mg, buprenorphine patches (5 mg patch), co‐codamol (codeine 50 mg, paracetamol 500 mg), vitamin C (1 g), vitamin D (800 units), cetirizine 10 mg, cream containing betamethasone and clotrimazole, omeprazole 20 mg, pregabalin 75 mg, testosterone 2% gel, clenil modulite 200 mcg inhaler twice a day. At the 6‐year visit, vitiligo had extended to all over his body including the covered areas, forehead, armpits, around his groin and his left hand. Topical steroid cream made no difference.

### 2.3 Case 3

**FIGURE 3:**
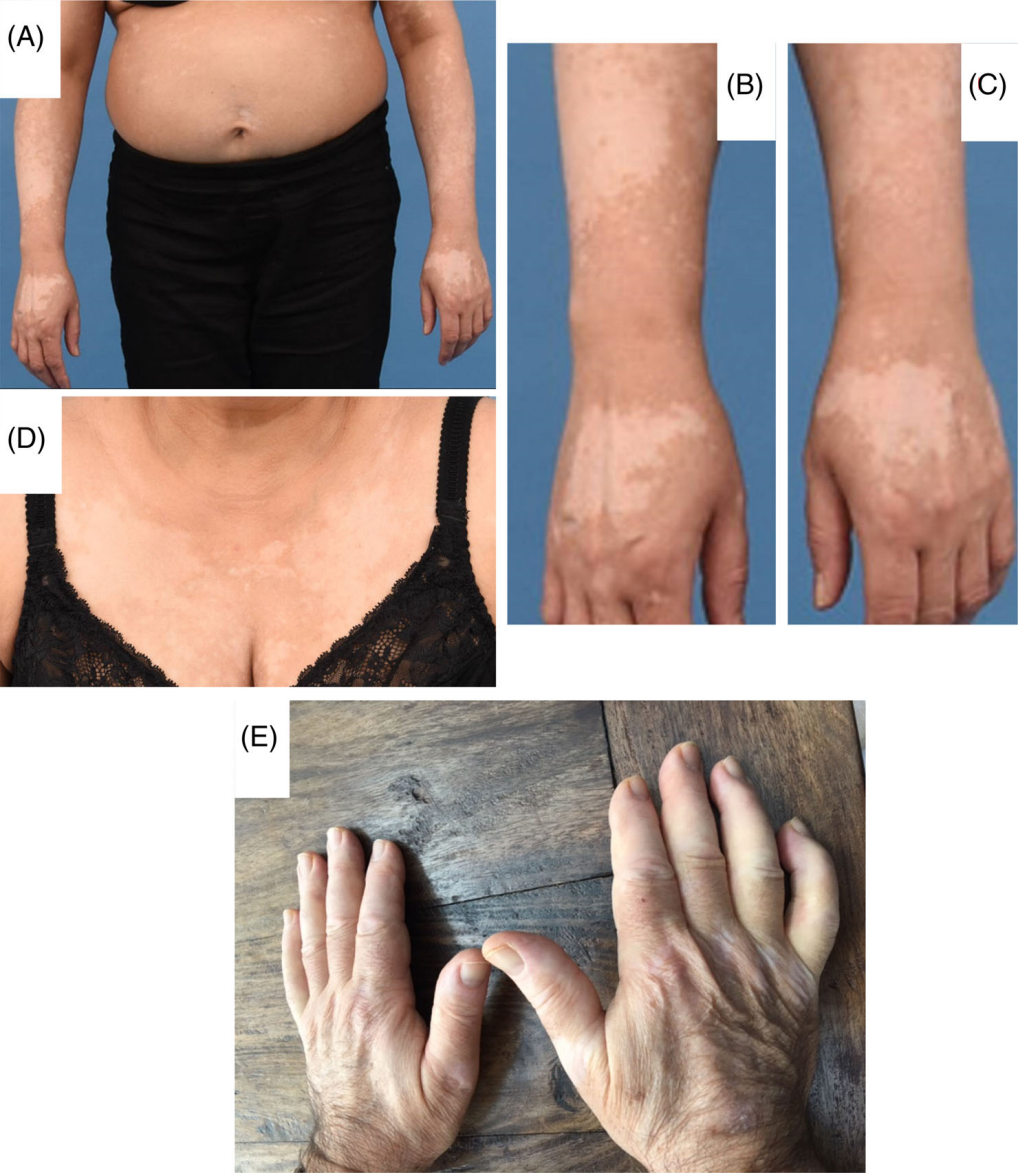
Photographs in case 3 shows vitiligo in 2018 on (A) trunk, (B) right hand, (C) left hand, and (D) front of chest wall. Photograph (E) shows vitiligo on hands in case 4

A 40‐year‐old woman of South Asian background, with AKU diagnosed at age 33 years, was referred for further management of AKU (Tables 1 and 2, Figure 3A‐D). She was born of a consanguineous union, and had a brother and six sisters, with a sister also suffering with AKU. She had ear and eye ochronosis, spondylosis (cervical, thoracic, and lumbar), and arthropathy (knees, shoulders). She has noticed change in the skin color especially around the eye and brown spots in the hands 3 years before the baseline visit. Her height and weight were 1.61 m and 75.6 kg, respectively, with a blood pressure of 110/77 mm Hg. Other health conditions included primary hypothyroidism requiring thyroxine therapy since age 18 years, and hypercholesterolemia managed by diet. Her medications at time of referral included tramadol 50 mg, meloxicam 7.5 mg, paracetamol 500 mg, thyroxine 100 μg, omeprazole, and calcichew D3 Forte (calcium 500 mg/vitamin D3 400 units). She was commenced on nitisinone 2 mg alternate days for 3 months and then the dose was increased to 2 mg daily. A year after the first visit, she noticed hyperpigmentary changes on her right forearm and both hands. At the 3‐year visit, she noticed depigmented macules were seen on her hands and anterior chest wall and a diagnosis of vitiligo was made. Vitiligo progressed further with pruritus. At her 5‐year visit, vitiligo also extended to her face with continued itching.

### 2.4 Case 4

A 67‐year‐old Caucasian man was seen in the Royal Liverpool University Hospital with documented AKU (Figure 3E, Tables 1 and 2). He had a brother and sister with AKU, and neither had vitiligo nor received nitisinone. He had advanced AKU features including bilateral ear and eye ochronosis, prostate stones, osteoporosis, mild aortic stenosis, ischemic heart disease, arthropathy (of left shoulder, left wrist, and both knees), as well as spondylosis (cervical, lumber, sacroiliac). His skin at baseline visit was normal. He was taking piroxicam 100 mg and potassium citrate at baseline. He started nitisinone 10 mg daily at his baseline visit. He had a successful coronary bypass surgery, 2 months after his baseline visit, following which he started taking omeprazole 40 mg, bisoprolol 2.5 mg, aspirin 80 mg, and rosuvastatin 10 mg, which he was on when he was seen at his three‐monthly visit after baseline. During his visit 3 months after baseline, he had vitiligo on his hands and feet, which persisted and progressed until discharge four from baseline. There was no personal or family history of autoimmune disorders. At discharge, he was receiving ibuprofen 400 mg, vitamin D 25000 units monthly, calcium carbonate 1.25 g, alendronate 70 mg, in addition to bisoprolol, aspirin, rosuvastatin, potassium citrate, and omeprazole, as drug therapy.

## 3 DISCUSSION

Eighty‐eight patients had attended NAC at least once; of these 28 had either not received nitisinone or not had a follow‐up visit on nitisinone. In these 88 patients, 29 were on proton‐pump inhibitor therapy (PPI), with 15 on lansoprazole and 14 on omeprazole. Vitiligo has not previously been described in AKU after nitisinone, even though there is a single case report of vitiligo in a 15‐year‐old boy with autoimmune diathesis and AKU.^16^ Four cases of vitiligo, three men and one woman, three with South Asian and one with Caucasian background, appearing at varying duration after beginning nitisinone treatment. None of the four patients had vitiligo at baseline pre‐nitisinone. These four cases all arose in adult patients over the age of 35 years, with the vitiligo showing a nonsegmental distribution with a predominant acro‐facial distribution. The prevalence of vitiligo has been reported around 1%.^17^ The prevalence of vitiligo in AKU patients treated with nitisinone attending the hospital from which this report appears is 5% (4 out of 80 nitisinone treated patients). This may suggest that nitisinone‐treated AKU patients may be at higher risk of developing vitiligo.

However, all four patients were also on PPI, at some point, now associated with development of vitiligo by poorly understood mechanisms. Case 1 was transiently on lansoprazole and only developed vitiligo 3 years later. Case 2 developed vitiligo more than a year after starting omeprazole. Case 3 was already on omeprazole at baseline and developed vitiligo 3 years after her baseline visit to the NAC. Case 4 developed vitiligo within weeks of starting omeprazole therapy. There is data describing omeprazole and its congeners inhibit melanogenesis in cells and human skin. It has been suggested that PPI could inhibit metallation of newly synthesized tyrosinase via ATP7A. The ATP7A protein regulates cellular copper homeostasis within cells thereby controlling the supply of copper to copper‐dependent enzymes, as well as the export of excess copper from the cytoplasm.^18^ The effect of PPIs appears to rapidly cause vitiligo, and only case 4 appears to fit the time‐line of starting omeprazole and appearance of vitiligo. Case 4 is also different from the other three cases in that the patient was Caucasian with no personal or family history of autoimmune diathesis. Since not everyone administered PPIs develop vitiligo, there is a suggestion that additional factor(s), currently unclarified, is(are) needed for the induction of vitiligo by PPI.^19^

Three of the patients had either autoimmune disease themselves or had a family history of autoimmune disease; vitiligo is an autoimmune destruction of melanocytes in the skin leading to depigmented macules. The two patients with personal autoimmune hypothyroidism had the most extensive vitiligo after starting nitisinone. Two of the four cases reported increased skin pigmentation before vitiligo appeared; following nitisinone serum and tissue tyrosine increase markedly, between 8.98 and 30.2 times baseline, making available more substrate for melanin synthesis. A previous NIH study has shown that nitisinone improved albinism due to increased melanin synthesis following the increase in tyrosine, demonstrating that increase in tyrosine circulating concentrations results in increased flux down the melanin pathway.^20^ Nitisinone‐induced transient hyperthyroidism occurred in a case with hypothyroidism on thyroxine replacement supporting the idea that increased tyrosine can drive pathways involving tyrosine (unpublished observations). Tyrosine administration has been shown to increase melanin formation.^21^, ^22^

Epidermal reactive oxygen species accumulation has been considered the main culprit in the pathogenesis of vitiligo, the most notable of which is hydrogen peroxide (H_2_O_2_). The concentration of H_2_O_2_ may reach a mmol/L.^23^ At this concentration, H_2_O_2_ leads to changes in the mitochondria and, consequently, apoptosis and death of the melanocytes.^24^ It has been shown that tyrosine upon entering the melaninogenic pathways, in which tyrosinases participate, produces certain electrically unstable by‐products, which have the potential to damage other cellular substrates resulting in death of the melanocytes.^25^

Three of the four cases also reported itching when the vitiligo appeared and continued over time. The exact mechanism of itching in vitiligo is not well understood. A recent review estimated the prevalence of itching in vitiligo at 20.2%.^26^ Nitisinone has been used in the treatment of children with HT‐1 since 1991 and the manufacturer's prescribing information does not list vitiligo as a side effect although it lists itching, urticaria, and maculopapular rash, all at a prevalence of 1%.^14^

**FIGURE 4:**
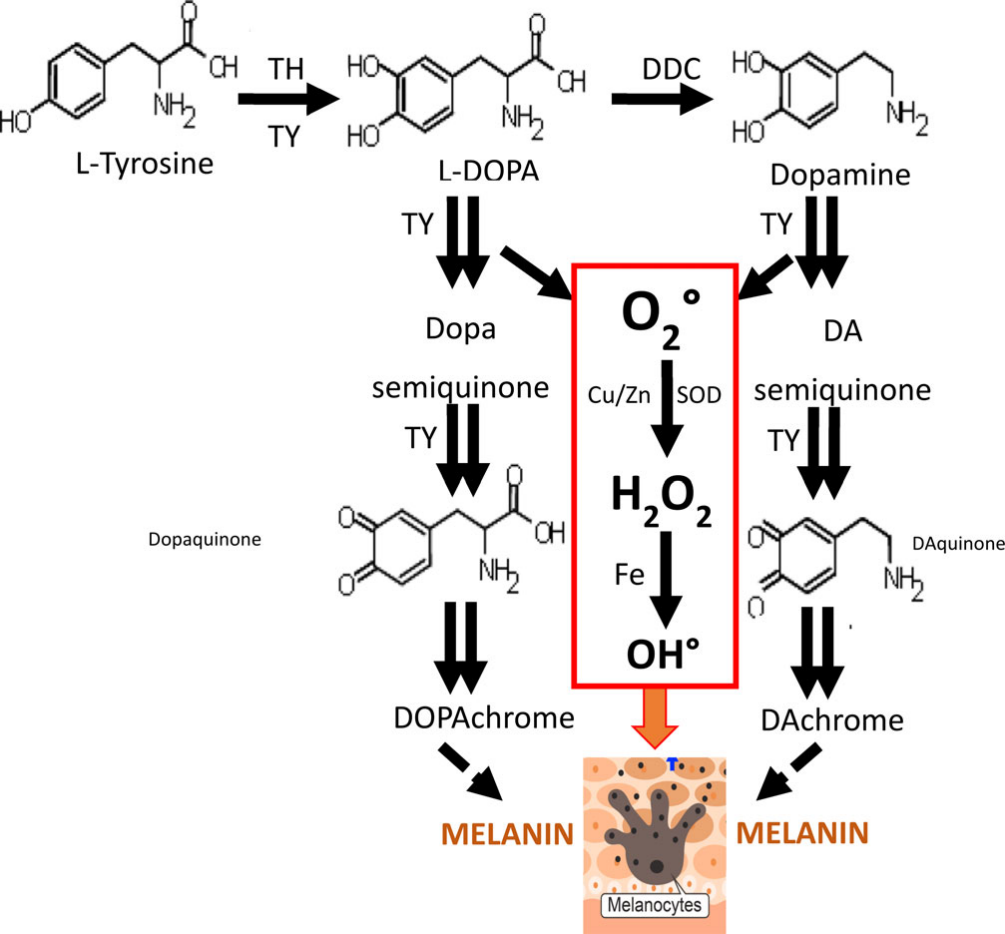
Tyrosine at normal concentrations is converted to melanin via parallel pathways of L‐DOPA and dopamine, mediated by tyrosinase, in the melanocyte. The action of tyrosinase results in reactive oxygen species (ROS) generation. ROS has been implicated in the death of melanocytes in the setting of autoimmune vitiligo. Homogentisic acid (HGA) as a reducing agent can protect against reactive oxygen species, which can be lost after nitisinone therapy, due to decrease in HGA. Further, tyrosine concentrations increase more than 10‐fold following nitisinone therapy, thus potentially increasing ROS generation by increasing flux in the melanin pathway, thereby further increasing the risk of melanocyte death. Cu, copper; DDC, dopamine decarboxylase; Fe, iron; H_2_O_2_, hydrogen peroxide; O_2_, superoxide; OH, hydroxyl radical; SOD, superoxide dismutase; TH, tyrosine hydroxylase; TY, tyrosinase; Zn, zinc

The mechanism underlying vitiligo in this case report is unknown. We speculate on the mechanism to explain the onset of vitiligo after nitisinone in these four AKU patients (Figure 4), as follows. In AKU, HGA is increased, as can be seen in all four patients at baseline, before commencement of nitisinone. HGA is a reducing agent and can mop up oxidant products including hydrogen peroxide; analytical interference due to this reducing property of HGA, in an enzymatic assay of creatinine has been previously described, and supports this role for HGA in AKU.^27^ Decrease in HGA after nitisinone could decrease this protection against oxidant stress, increasing the vulnerability to vitiligo. Additionally, following administration of nitisinone, the inhibition of 4‐hydroxyphenylpyruvate dioxygenase results in a marked increase in serum and tissue tyrosine.^28^ Since the usual metabolism of tyrosine through HGA is interrupted, increased flux along alternate pathways results. One of these alternate pathways is the melanin synthesis pathway, leading to stimulation of melanin formation, with hyperpigmentation, noted in two of the four patients, supports this idea. Increased oxidant reactions during stimulation of melanin formation lead to production of oxidants including hydrogen peroxide, which has been linked with melanocyte damage and cell death.^23^, ^24^, ^25^ We also propose that the decrease of HGA and the increase in tyrosine after administration of nitisinone in AKU patients accelerate underlying autoimmune diathesis, predisposing to the melanocyte death and appearance of vitiligo. Support for this hypothesis comes from the fact that three of the four patients had autoimmune disease or a family predisposition to autoimmune disease. The decrease in HGA and the increase in tyrosine can be readily appreciated from Table 1. It is possible that UV radiation from sunlight could participate in the vitiligo process as all four patients experienced vitiligo in the hands and face, exposed to sunlight, before spreading further. The greater frequency of vitiligo in patients with South Asian background in this report, three of four patients, who have an increased activity of the melanin pathway, compared with Caucasians, is consistent with our hypothesis. It has been shown that more pigmented skin can produce more melanin when stimulated such as by UV radiation of sunlight, with response in South Asians four times greater than in white Caucasians.^29^, ^30^ The quickest appearance of vitiligo following nitisinone administration was in case 4 who received the larger dose of nitisinone, and also with the greatest increase in serum tyrosine. The final observation that only one of the four patients in this report was a woman, generally more prone to autoimmune disease, could be due to the beneficial effect of estrogen in directing the tyrosine pathway toward 4‐hydroxyphenylpyruvate through stimulation of rate‐limiting tyrosine aminotransferase.^31^

Finally, nitisinone was continued in these four patients because of the lack of certainty about causation. Further, it is the only treatment option for the progressive severe morbidity, which would result if nitisinone was withdrawn in these cases without any other HGA‐lowering disease‐modifying therapy on the horizon. Vitiligo is generally considered irreversible; it is a dilemma whether to stop nitisinone knowing that vitiligo will probably not reverse, but with the certainty that AKU disease will progress. It is important that patients be counseled about this risk when it appears, and involved in the decision to continue or stop nitisinone. It is also worthwhile bearing in mind that other medications, such as PPI, could not only independently cause vitiligo but could possibly enhance or add to the risk of vitiligo due to nitisinone and/or autoimmune diathesis; alternatives to PPIs in the context of nitisinone in AKU especially in the setting of autoimmune disease predisposition should be considered. Further investigations are needed addressing the risk of vitiligo in nitisinone therapy in those with a personal or family history of autoimmune diseases. We are learning more about nitisinone therapy in adult AKU and we should be vigilant in recognizing new health issues in these situations going forwards, with nitisinone therapy now approved for the treatment of adult AKU by the European Medicines Agency.^32^

**Learning points**

- Vitiligo may be more prevalent in AKU after nitisinone therapy.
- AKU patients with pigmented skin may be more at risk of vitiligo after nitisinone.
- Itching may be an accompanying factor.
- Personal or family history of autoimmune disease may increase risk of vitiligo in AKU post‐nitisinone.
- Use of other vitiligo‐causing medications should be reviewed to minimize vitiligo after nitisinone.

## CONFLICT OF INTEREST

The authors declare no potential conflict of interest.

## AUTHOR CONTRIBUTIONS

Lakshminarayan Ranganath assessed the patients, recognized the adverse event as being related to nitisinone, and wrote the manuscript. Milad Khedr assessed the patients, and edited the manuscript. Leanne A. Evans was involved in compiling the data and edited the manuscript. Helen Bygott suggested the omeprazole link to vitiligo, and edited the manuscript. Emily Luangrath helped compile the data and edit the manuscript. Elizabeth West helped assess the patients and edited the manuscript.

## ETHICS STATEMENT

The data collected from the NAC for patients 1 to 3 were approved by the Institutional Audit Committee (Audit No: ACO3836). Patient 4 with alkaptonuria participated in the Suitability of Nitisinone in Alkaptonuria 2 (SONIA 2) study (EC Liverpool [NRES Committee North‐West ‐ Liverpool Central] Reference number: 13/NW/0567).

## INFORMED CONSENT

All procedures followed were in accordance with the ethical standards of the responsible committee on human experimentation (institutional and national) and with the Helsinki Declaration of 1975, as revised in 2000 (5). Informed consent was obtained from all patients for being included in the study. Further clarification has been provided in the previous page.

## Data Availability

The authors agree to honor any reasonable request by other researchers for materials, methods, or data necessary to verify the conclusion of the article.
